# Clinical Findings in Patients With Persistent Positional Nystagmus: The Designation of “Heavy and Light Cupula”

**DOI:** 10.3389/fneur.2019.00326

**Published:** 2019-04-09

**Authors:** Xiaowu Tang, Qiuhong Huang, Ling Chen, Peng Liu, Tianci Feng, Yongkang Ou, Yiqing Zheng

**Affiliations:** ^1^Department of Otolaryngology-Head and Neck Surgery, Sun Yat-sen Memorial Hospital, Sun Yat-sen University, Guangzhou, China; ^2^Institute of Hearing and Speech-language Science, Sun Yat-Sen University, Guangzhou, China; ^3^Xinhua College Sun Yat-Sen University, Guangzhou, China

**Keywords:** DCPN, light cupula, heavy cupula, null plane, reposition maneuver

## Abstract

**Objective:** Direction-changing positional nystagmus (DCPN) had been observed as persistent horizontal apogeotropic and was considered as “cupulolithiasis or heavy cupula. ” Recently, the concept of “light cupula” exhibiting persistent geotropic DCPN has been introduced. However, the light cupula is not systematically described, while the identification and diagnosis of “light cupula” should be improved. Here we investigated the underlying characteristics and therapeutic options designed to the “light” and “heavy” cupula, respectively; and summarized the clinical characteristics and therapeutic effect in the two groups.

**Methods:** A total of 359 cases with vertigo and bilateral DCPN were found in the supine roll test. Only 25 patients with persistent DCPN were enrolled and followed up. According to the direction of nystagmus, we further divided the patients into “heavy cupula” (apogeotropic) and “light cupula” (geotropic) groups. We compared the incidence, characteristics of nystagmus and the efficacy of repositioning maneuver in the two groups.

**Results:** Nine patients with persistent horizontal geotropic DCPN were confirmed as “light cupula,” other 16 patients with persistent horizontal ageotropic DCPN were confirmed as heavy cupula. All 25 patients had null plane; the mean value and standard deviation of the null plane in light cupula and heavy cupula was 25.67 ± 9.31° and 27.06 ± 6.29°, respectively. The mean value and standard deviation of the termination plane in light cupula was 28.78 ± 10.00°, and 30.25 ± 6.53° in heavy cupula. There was no statistical significance between the two groups. We found that the direction of evoked nystagmus in the supine position was toward the intact side in light cupula, while in heavy cupula, it was toward the lesion side. The null plane appeared on the lesion side. For light cupula patients, the effect was not obvious at Day-7 after the treatment, however, treatment for most heavy cupula patients were effective. All patients recovered after 30 days of treatment.

**Conclusion:** The null plane is crucial in determining the lesion side for light or heavy cupula. Although the short-term therapeutic effect of the light cupula is not as promising as the effect seen in heavy cupula, the long-term prognosis in both groups is comparable; with all patients recovered after 30 days of treatment.

**Study design:** This is a retrospective cohort study.

## Introduction

Benign Paroxysmal Positional Vertigo (BPPV) is the most frequent episodic vestibular disorder. The BPPV horizontal semicircular canal recognizes two variants, conductolitiasis and cupulolithiasis, with the former being more frequently diagnosed (1). It has been reported that approximately 10–30% of BPPV originate from the horizontal semicircular canal (2). For patients with LSCC BPPV, direction-changing positional nystagmus (DCPN) is typically observed in a supine roll test. Moreover, LSCC BPPV can be divided into horizontal apogeotropic DCPN and geotropic DCPN. Generally, if the apogeotropic DCPN is persistent and lasts for more than 1 min, it is considered as cupulolithiasis or heavy cupula where the otolith debris may attach onto the cupula. In the clinics, the duration of the most of evoked geotropic nystagmus was less than 35 s and relatively gradually weakened or disappeared after positional examination, which is commonly considered as canalithiasis.

However, recently, a new concept of “light cupula” exhibiting persistent geotropic DCPN has emerged (3). “Light cupula” accounts for special geotropic positional vertigo where the geotropic DCPN is always persistent and lasts for more than 1 min but lacks latency or fatigability. Although the positional vertigo and nystagmus of light cupula manifests similarly to BPPV, whether or not it is a special type of BPPV is yet to be determined, and its pathogenesis is still unclear and is generally considered as a vestibular pathological phenomenon and theoretical hypothesis corresponding to clinic (4). In light cupula, the specific gravity of the cupula is lower than that of the surrounding endolymph, which either activates or inhibits hair cells under the cupula according to the head position in the gravitational plane (3, 5). Ichijo et al. hypothesized that the buoyancy traction migration of the ampulla ridge was caused when the low-density debris adhered to the ventral ridge of the affected side of the ampulla (6).

More interestingly, in both light cupula and heavy cupula, symptoms of patients with static DCPN disappeared on a certain plane when the head was rotated to a certain degree from the supine position. Therefore, this point is fixed as the null plane(7). The null plane, also known as “zero plane,” refers to the certain plane where the nystagmus disappeared during the head turning from side to side.

The null plane is the most important characteristic for the diagnosis of light cupula. However, to date, the underlying pathogenesis of light cupula is unknown, and the standard of diagnosis and identification of this disease are confusing in clinical practice, with no available effective treatment options.

In this paper, we intend to explore the possibility in exacting the null plane in precise diagnosis of the two types of DCPN in order to suggest a standardized diagnosis and treatment option for both heavy and light cupula.

## Patients and Methods

### Patients

In this study, a total of 359 cases of bilateral DCPN were found in the supine roll test in patients with vertigo who visited the outpatient department of Sun Yat-sen Memorial Hospital, Sun Yat-sen University from July 2016 to May 2017. All the patients' symptoms were recorded by Vesticon system 2000 infrared video ophthalmogram recording analyzer Videonystagmography (VNG) during the supine roll test. Spontaneous nystagmus was recorded in the sitting position both with and without visual fixation. Gaze-evoked nystagmus, and horizontal saccades and smooth pursuit were also evaluated.

From the 359 cases of bilateral DCPN, we analyzed the “heavy cupula” and “light cupula” patients according to the following parameters of nystagmus (Tables 1, 2).

**Table 1:** Inclusion criteria of light cupula.

(1) No nystagmus observed in the sitting position
(2) Presence of horizontal persistent nystagmus in the supine position
(3) Horizontal geotropic DCPN in the Supine Roll test
(4) The duration of DCPN is more than 1 min
(5) No latency or fatigability observed
(6) Presence of null plane


**Table 2:** Inclusion criteria of heavy cupula.

(1) No nystagmus observed in the sitting position
(2) Presence of horizontal persistent nystagmus in the supine position
(3) Horizontal apogeotropic DCPN in the Supine Roll test
(4) The duration of DCPN is more than one min
(5) No latency or fatigability observed
(6) Presence of null plane

All patients had audiology and imaging examination. Audiology examination showed no obvious abnormality in all patients. Brain MRI was supplemented in all patients to exclude other vestibular and neurological diseases (8). The Dix-Hallpike test was routinely performed simultaneously to exclude other positional nystagmus. No drugs affecting central nervous system excitability or inhibition was taken at least 24 h prior to examination.

All patients agreed to participate in the study were given a thorough explanation of the study and have given their written consent. The patients were followed up until the completion of the study. This study was approved by the Ethics Committee of Sun Yat-sen Memorial Hospital, Sun Yat-sen University.

## Method

### Examination Procedure and Nystagmus Analysis

**Figure 1:**
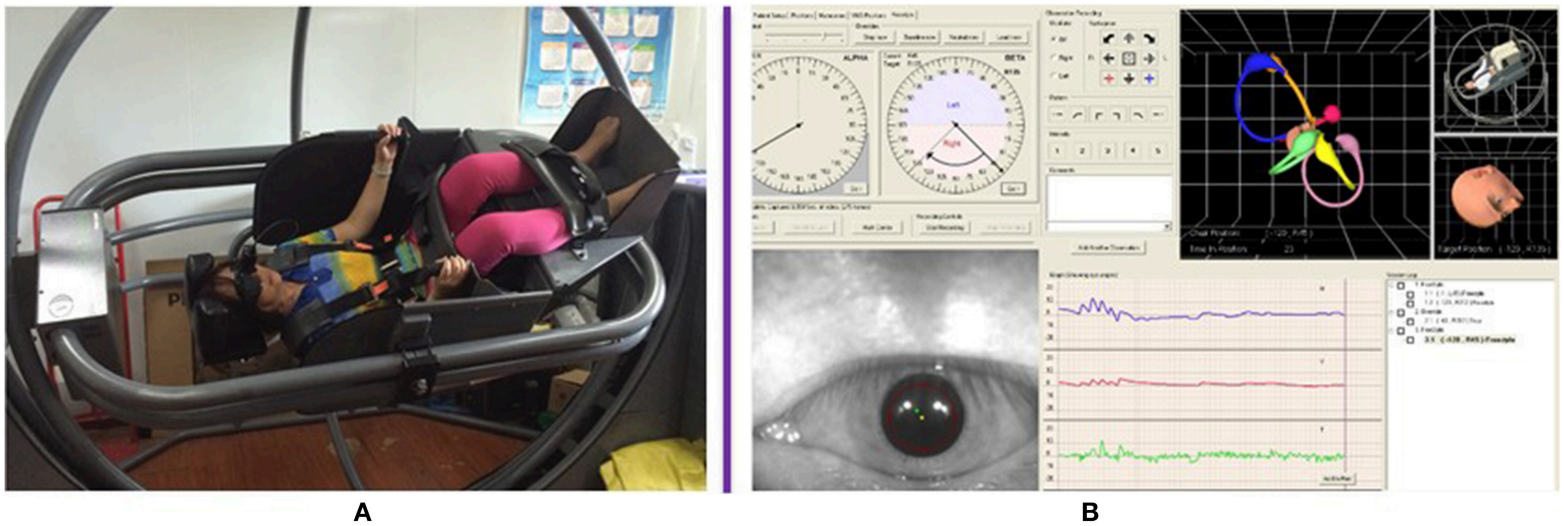
Automatic rotary instrument Epley Omniax360 showed the position of the patient and the exact angle when body rotation. **(A)** The position of the patient. **(B)** The exact angle when body rotating from one plane to another as seen on the computer software.

The presence of horizontal DCPN was analyzed during the running of a series of head positions using Epley Omniax360, which is designed as automatic rotary instrument for the positional test and repositional maneuvers. The instrument can be used for head and body fixation of 360°. The software simulates the deflection angle of the head- semicircular canal on different planes for measuring and recording (Figure 1).

A series of head positions were done in the following order: U (sitting, head in normal position and straight forward); S (supine position, head elevated 30°); SL (supine position and then head turned max to the left); SR (supine position and then head turned max to the right); DHL (hanging head 45° to left); DHR (hanging head 45° to right); The evoked nystagmus were recorded using EPLEY OMNIAX360, and digitally stored in degrees per second. Spontaneous, positional, latency and duration of position-induced nystagmus were recorded. The nystagmus was observed for at least 2 min at each position, the maximal SPV was compared between turning to the right and left during the supine roll test.

Null plane: by slowly rotating the subject's head in the S position from left ear down to right ear down, a plane was sought where there was no vertigo and nystagmus and beyond which the nystagmus changed direction.

### Treatment

**Figure 2:**
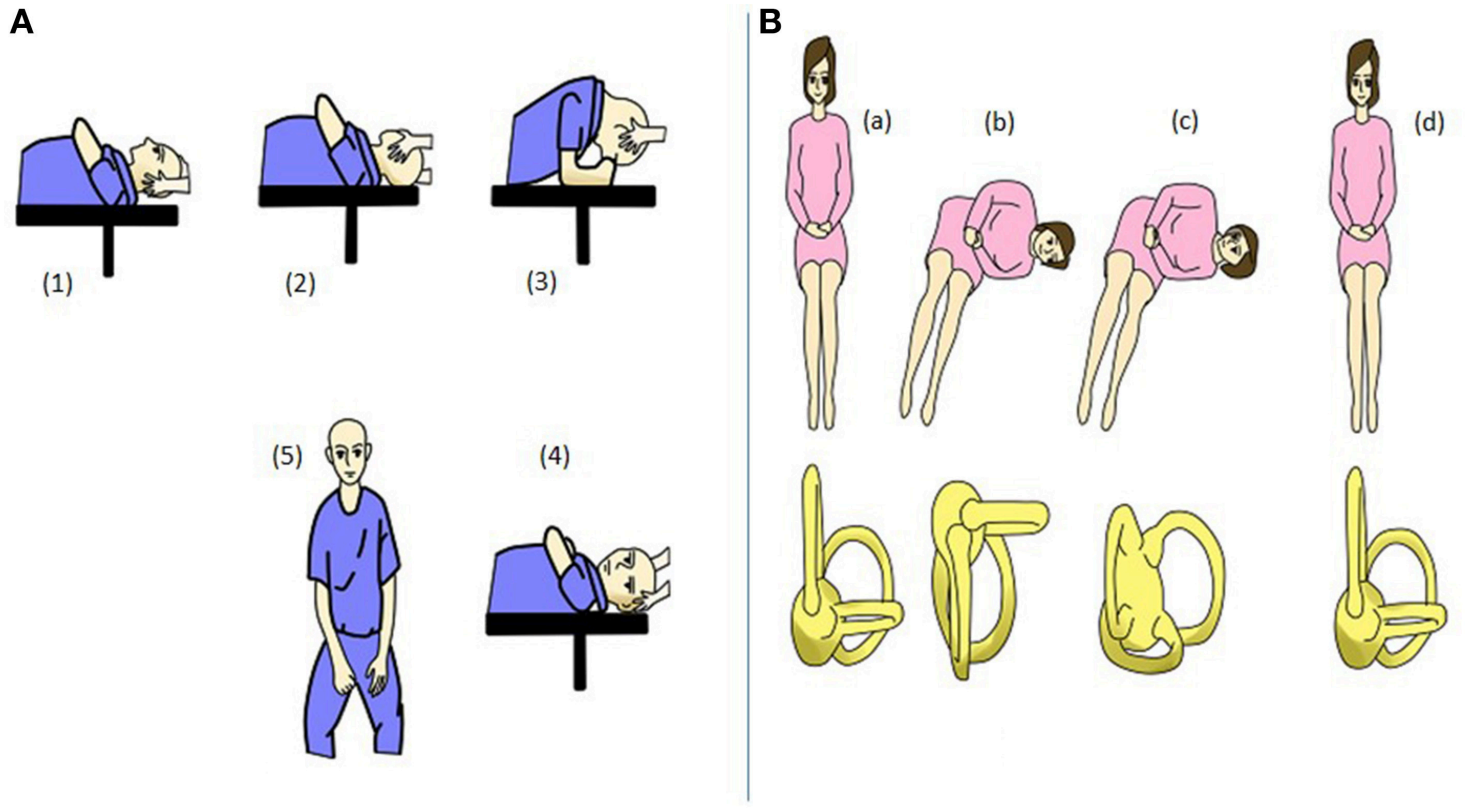
Repositional maneuvers. **(A)** Barbecue maneuver. **(1)** Lie flat on back from an upright position. **(2)** Turn 90° to left (or right) along the longitudinal axis body. **(3)** Continue to turn 90° in the same direction. **(4)** Continue to turn 90° in the same direction. **(5)** Sit upright. **(B)** Gufoni maneuver. **(a,b)**. Lie on affected side rapidly from upright position. **(c)** Rotate the head 45° quickly to the contralateral side. **(d)** Sit upright.

The repositioning maneuvers to treat the patients with light cupula was done using the Barbecue method. In brief, Barbecue was performed as: (1) lie flat on the back from an upright position (2) turn to the left (or right) along the longitudinal axis body turn 90° (3) continue to turn to the same direction of 90 °(4) continue to turn again to the same direction of 90°(5) sit upright (Figure 2A).

The patients with heavy cupula were successfully converted to geotropic nystagmus by the Gufoni method. Gufoni was performed as: (1) the patient is sitting, rapidly lying on his or her affected side (2) head is quickly turned to the contralateral rotation 45° (3) sit upright, and then continue with by Barbecue method (Figure 2B).

Position tests were re-evaluated weekly after the repositioning maneuver. If positional vertigo still persisted in patients 1 week after treatment, the Barbecue method was performed again, with the supplementation of the Gufoni method if necessary. All patients were followed up every week and followed up for more than 1 month. The therapeutic effect of the repositioning maneuvers was evaluated at Day-7 and Day-30.

### Statistical Analysis

SPSS19.0 statistical software was used for all data analysis. *T-*test was used for the comparison between two groups. All data were considered statistically significant if *p* < 0.05.

## Results

### Characteristics of Nystagmus

From 359 patients, after considering the inclusion and exclusion criteria, we finally recruited 25 patients with persistent horizontal DCPN (25/359, 4.6%). The course of the patients lasted 1 to 13 weeks, among which 21 cases were primary and 4 cases were secondary, including three cases of sudden deafness and one case of Meniere's disease. Gender wise, 7 patients were male with the average age of 50.4 ± 14.7, and 18 female patients with the average age of 47.9 ± 7.

Of the 25 patients, 9 (3 males and 6 females) showed persistent geotropic DCPN with a null plane during the supine roll test and were diagnosed with light cupula; 16 (4 males and 12 females) showed persistent apogeotropic DCPN with a null plane and were diagnosed with heavy cupula. The mean value and standard deviation of the occurrence and termination angle of the null plane were recorded and shown in Table 3.

**Table 3:** Null plane occurrence and termination angle of 25 patients with horizontal DCPN(°).

**Null plane**	**Light cupula**	**Heavy cupula**	**t**	P^*^
Occurrence angle	25.67 ± 9.31 (θ_1_)	28.78 ± 10.00 (β1)	0.109	2.782
Termination angle	27.06 ± 6.29 (θ_1_ + θ_2_)	30.25 ± 6.53 (β1 + β2)	0.086	3.226

* *No statistically significance between the two groups(P > 0.05)*.

**Figure 3:**
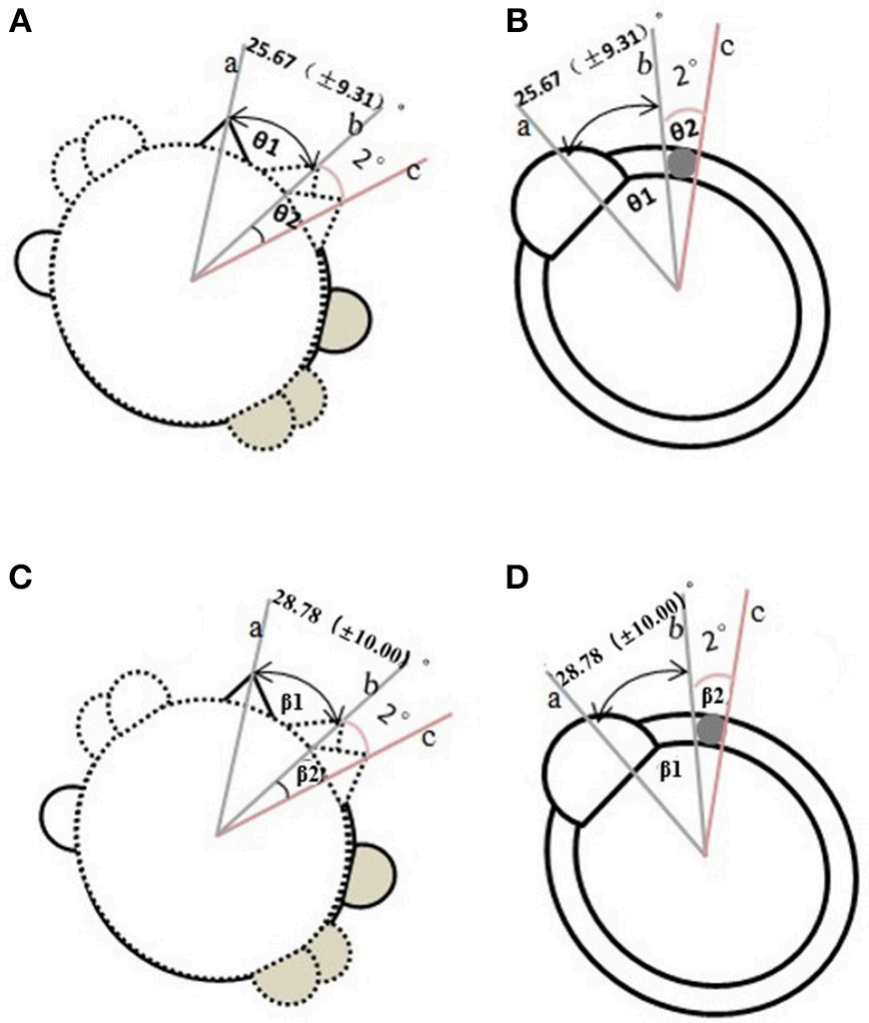
Determination of the null plane in light cupula and heavy cupula. **(A)** The method in determining the null plane in the light cupula (view of head rotation). (A-a) the supine position. (A-b) the plane from the supine position turning to the affected side when the evoked-DCPN disappeared. (A-c) another plane that continued turning the head to the affected side when the evoked- DCPN re-appeared. (θ1 was the angle from line a to b. θ2 was the angle from b to c, termed as the null plane). **(B)** The view of semicircular canal corresponding to **(A)**. **(C)** The method in determining the null plane in the heavy cupula (view of head rotation). (C-a) The supine position. (C-b) the plane from the supine position turning to the affected side when the evoked-DCPN disappeared. (C-c) Another plane that continued turning the head to the affected side when the evoked-DCPN re-appeared. (θ3 was the angle from line a to b. θ4 was the angle from b to c, termed as the null plane). **(D)** The view of semicircular canal corresponding to **(C)**.

In our study, we found that the rotational angle from the ceasing of nystagmus to re-appearance was about 2° (Figure 3) and was designated as null plane. Vertigo and nystagmus also disappeared when the head was rotated to 180° from the null plane.

In the group of light cupula diagnosis, the stronger side of geotropic nystagmus (lesion side) on supine roll test was identified in 6 of 9 patients (67%). The null plane of the 6 patients all appeared on the side of the lesion. We were unable to identify the lesion side with supine roll test in the remaining 3 cases. In the group of heavy cupula diagnosis, the weaker side of apogeotropic nystagmus (lesion side) on supine head roll test was identified in 12 of 16 patients (75%). The null plane of the 12 patients were all on the side of the lesion. We were unable to identify the lesion side with supine roll test in the remaining 4 cases.

Molina, MI (9) reported that Canal paresis (CP) was observed in 25% (16/64) of cases with BPPV. In our study, vestibular function examination: Due to the requirements of patients' wishes, 15 in 25 patients underwent caloric testing after the complete disappearance of positional nystagmus (5 cases with heavy cupula and 10 cases with light cupula). The results showed that 0 of 5 cases with heavy cupula had CP, while 2 of 10 cases with light cupula (20%) had CP, which was the same result reported by Hiroaki Ichijo (10).

In addition, we found that the direction of evoked nystagmus in the supine position in light cupula was toward the intact side, while in heavy cupula, the direction of evoked nystagmus in the supine position was toward to the lesion side. We also observed that direction of evoked nystagmus in the supine position in light cupula was in the contralateral side of the null plane location, while the direction of evoked nystagmus in the supine position in heavy cupula was at the same side of null plane. Therefore, another simple yet reliable method of differentiating these two groups is to determine the location of the affected area with a reference to the null plane.

### Efficacy of Repositioning Maneuver Therapy

**Figure 4:**
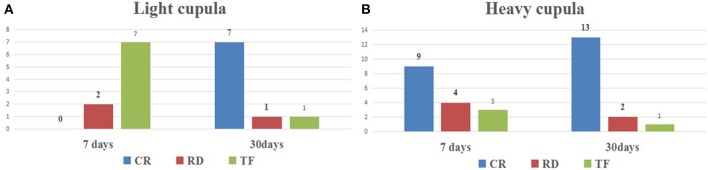
The therapeutic effect of repositioning maneuver in the light cupula and heavy cupula. **(A)** The effect was evaluated at Day-7 days and Day-30 after repositioning maneuver in heavy cupula. **(B)** The effect was evaluated at Day-7 days and Day-30 after repositioning maneuver in light cupula. (CR, complete resolution; RD, residual dizziness; TF, treatment failure).

According to the intensity of nystagmus, the side of affected ear was determined. In the light cupula group: 2 cases were on the left; 4 cases were on the right; and in heavy cupula group: 5 cases were on the left and 7 cases were on the right. The patients in both groups were treated with repositioning maneuver (using Barbecue, Gufoni and Barbecue method as described above). The therapeutic efficacy of this method was determined at Day-7 and Day-30 (Figure 4). The outcome was categorized as either complete resolution, partial/no resolution, or canal conversion. For those who were categorized as partial/no resolution or canal conversion, a follow up visit was arranged within 7 days, and these patients were treated with another cycle of appropriate procedure (as mentioned in Methodology). Successful repositioning was defined as complete resolution (CR) of any positional nystagmus. Residual dizziness (RD) was defined as a subjective report of partial or no response despite successful repositioning. Treatment failure (TF) was defined as partial/no resolution after four or more cycles of repositioning (11).

Since the intensity of nystagmus could not be used to determine the lesion side for some cases, t, the side of null plane was used to determine the side of lesion. The results showed that in light cupula group 2 cases were on the left, 1 case was on the right; and in heavy cupula group 3 cases were on the left and 1 case was on the right.

Seven days after the repositioning maneuvers of the light cupula, only 2 cases showed improvement (2/9; 22.2%), and the other 7 patients still complained of nystagmus and vertigo. 30 days after the repositioning maneuvers, all the light cupula patients were fully cured. Whereas, in the heavy cupula group, 81% (13/16) showed improvement 7 days after the repositioning maneuvers, with disappearance of vertigo and nystagmus. Position test were re-evaluated after 30 days, and the results indicated that only 1 case (6.3%) reported no improvement (Table 4), which indicated that the repositioning maneuvers were effective for the treatment of heavy cupula, while affirming the fact that the side of lesion could be correctly determined by using the null plane. The treatment effect of light and heavy cupula is consistent with the report by Hiruma et al. (12). Therefore, with this method, patients with cupulopathy would now have a better long-term prognosis.

**Table 4:** The therapeutic effect of repositioning maneuver in the light cupula and heavy cupula during follow-up after 7 days and 30 days.

	**CR**	**RD**	**TF**
**7 DAYS-FOLLOW UP**			
Light cupula	0	2	7
heavy cupula	9	4	3
**30 DAYS-FOLLOW UP**			
Light cupula	7	1	1
Heavy cupula	13	2	1

*CR, complete resolution; RD, Residual dizziness; TF, Treatment failure*.

## Discussion

Cupulolithiasis or “heavy cupula” was diagnosed when the duration of position-induced horizontal apogeotropic DCPN is persistent for more than 1 min. As the otolith debris is attached onto the cupula, the sensitivity of the cupula increases when gravity changes. The formation of “heavy cupula” (7) was relative to the endolymphatic density. In a few of patients with apogeotropic DCPN, persistence within 1 min was thought to be caused by otolith flow to the anterior part of semicircular canal of the ampulla, which is also considered to be canalithiasis (13). However, canalithiasis was most commonly seen as geotropic DCPN for no more than 35 s, which the otolith debris may be located in the long arm of LSC. Recently, we found another type of persistent horizontal geotropic DCPN in the clinics, with more than 1 min persistence without latency or fatigability, which is defined as “light cupula.” In 1956, Aschan et al. (14) described the nystagmus, which was called alcoholic nystagmus, a symptom appearing after drinking. It was found that this type of positional vertigo was similar to the horizontal semicircular cupulolithiasis, but it could not be explained by the theory of canalithiasis. Bergenius et al. (3) further proposed that the name “light cupula nystagmus” and further described its characteristics. The present study suggests that light cupula is not a disease but is a vestibular pathological phenomenon and theoretical hypothesis corresponding to BPPV pathogenesis.

The common characteristics observed in patients with light or heavy cupula where head position-induced persistent horizontal DPCN in the Supine roll test (2) are (i) Persistent horizontal nystagmus with fixed orientation in either lying, supine or prone position; (ii) symptoms that last for more than 1 min; (iii) no latency and fatigue observed; and (iv) the presence of null planes (15). We found that not all horizontal semicircular canal BPPV patients in supine position will have sustained fixed nystagmus and null plane. Whether this result is dependent on the location of otolith adhesion or not remains to be further studied. On the basis of a morphological study of squirrel monkeys and computed tomography (CT) of the human inner ear (7), the top of the horizontal canal cupula is leaning toward the lateral side, and therefore we can estimate that the angle between the sagittal plane and the horizontal canal cupula is approximately 20°. When the head is in bowing or leaning position, it further deflects the head toward the lesion side. This would lead to the formation of a parallel axis of the plane of gravity and the ampulla of the lesion. When the ampulla no longer deviates from the utricle, the nystagmus stops. This point is dictated as the null plane.

Previously, Kim et al. (16) and Ichijo et al. (7) reported that Supine roll test based on the head deflecting can be used to determine the null plane. Furthermore, Kim (15) reported that the head bending and elevation also could be used, which was similar to the Bow and Lean Test (BLT). He hypothesized that the nystagmus was evoked when the patient was sitting upright both in heavy and light cupula. The first null plane is determined when the nystagmus disappeared on the certain position when the head was bowed or leaned at a certain angle. Choi et al. (17) reported that the BLT were observed in 65% (48 of 74) of patients with LSCC canalolithiasis. In his study bowing nystagmus (BN) and leaning nystagmus (LN) were in the opposite direction in 38 (out of 48) patients and in the same direction in 10 (out of 48) patients. In our current study, the direction of BN by BLT was opposite to the LN in all patients. In the heavy cupula patients, BN was directed toward the intact side, and that LN was directed toward the lesion side. Vice versa, in the light cupula patients, BN beat toward the lesion side,and that LN beat toward the intact side.

When the head was supine and hanged 60°, then the nystagmus was evoked, while nystagmus symptom disappeared when the head was turned either to the left or right at 20°-30°. This is determined to be the second null plane. In our study, we did not find the evoked horizontal nystagmus that was previously reported by Kim et al. (16) in the sitting upright position, which was pseudo spontaneous nystagmus. It is speculated that there is a 30° difference between the upright head LSC and the horizontal plane, putting the ampullary at a higher position than the other semicircular canals, which in turn cause the light or heavy cupula to either deviate or move closer to the utricle (2). We also found that it was more difficult to find the null plane with the BLT method in clinical practice than that with the Supine Roll test, and it was hard to accurately measure the angle of head bend forward and backward. In this study, we found that nystagmus could be evoked in all the 25 patients on the supine position (Although in light cupula, the nystagmus was directed to the intact side, while in heavy cupula, the nystagmus was directed to the lesion side), while continued turning of the head at a 10°-38° to the lesion side could relieve the nystagmus syndrome. This null plane agrees with the angle reported by Kim et al. (13).

Kiyoshi Hiruma et al. (12) used infrared photogrammetry or Frenzel glasses and a protractor to measure the null plane angle in the heavy cupula or light cupula. Although this method was comparatively easy, the correct posture of patient was too difficult to control. Here we used the EPLEY OMNIAX automatic positional vertigo diagnosis instrument (Vesticon company, USA), the angle is directly displayed on the computer software along the angle of the head—body axis plane, which can be displayed and measured accurately by the computer. Our results showed that there was no significant difference in the occurrence and termination angle of null plane between the light cupula and the heavy cupula.

According to the Committee of the Bárány Society in 2017 (2), the diagnostic criteria for BPPV are as follows: In the supine roll test, patients with geotropic nystagmus usually display stronger intensity—when the head turned to the affected ear, while in patients with apogeotropic nystagmusis, stronger intensity was observed when the head turned away from the lesion ear. In this study, we had 25 patients with persistent DCPN. Among them, there were 9 cases with light cupula and 16 cases with heavy cupula. In the light cupula group, the remaining 3 cases were unable to identify the lesion side with supine head-roll test, while in the heavy cupula group, there were 4 cases. In the process of null plane detection, we found that if nystagmus direction in the supine position was consistent with the null plane side, which was heavy cupula, and on the contrary, was light cupula. The null planes all appeared on the lesion side—this can be mutually authenticated with Supine roll test. In the vestibular function examination, CP did not appear in the heavy cupula group during the caloric testing, while 20% of the light cupula group had CP on the lesion side.

The patients with heavy cupula were treated with repositioning maneuvers. After Gufoni maneuvers (10 cases), 62% cases were converted into geotropic nystagmus, and then were further treated with the Barbecue method. The efficacy rate of short-term (1 week) and long-term (1 month) were 75 and 94% respectively. Otoliths in the cases with heavy cupula successfully converted may adhered to the ampulla rather than the utricle. Therefore, we thought that null plane can help the light and heavy cupula to determine the lesion side. Accurate localization depends on the efficacy of repositioning maneuvers.

The success rate of light cupula repositioning maneuvers is low, which corresponds to the result reported by previous studies (12, 15). Park et al. (18) reported that intratympanic steroid injection can be used in light cupula, however, there was no significant different at 1 week follow-up when comparing to the other treatment, perhaps due to the unclear pathophysiology of the light cupula. To date, there are three major hypotheses of the above phenomenon: (1) a light cupula is caused by a decrease in the density of the cupula (14) (2) an increase in the specific gravity of the endolymph leads to a decrease in the relative density of the cupula (6, 12) (3) light debris attached to the cupula gives rise to a light cupula (3). In support of the last hypothesis, Kim et al. (16) suggested the possibility that light debris were water-soluble macromolecules, such as proteoglycans, in the endolymph (16).

## Limitations

Caloric tests were only performed in 10 patients due to the wishes of patients. Although the video impulse test is known to be more sensitive to define vestibular function, we could not carry out this experiment due to the lack of instrument.

## Conclusion

Taken together, this study elucidated a simple yet accurate method in determining light and heavy cupula; depending on the side of supine position and null plane. Same-side supine position and null plane indicated heavy cupula while opposite-side supine position and null plane indicated light cupula. Although it is known that the side of null plane is always located on the side of lesion, in some cases, the lesion side may not be easy to determine, as it is difficult to be detected via nystagmus intensity. In this situation, the null plane is a very effective method to identify the lesion side of light cupula or heavy cupula. Moreover, the caloric testing is also another way for light cupula to identify the lesion side.

## Author Contributions

XT and QH analyzed the data and drafted the manuscript. LC, PL, and TF collected candidate information. YO and YZ edited the manuscript.

### Conflict of Interest Statement

The authors declare that the research was conducted in the absence of any commercial or financial relationships that could be construed as a potential conflict of interest.
